# The p53 Family: A Role in Lipid and Iron Metabolism

**DOI:** 10.3389/fcell.2021.715974

**Published:** 2021-07-29

**Authors:** Kyra Laubach, Jin Zhang, Xinbin Chen

**Affiliations:** Comparative Oncology Laboratory, Schools of Veterinary Medicine and Medicine, University of California, Davis, Davis, CA, United States

**Keywords:** p53, p63, p73, metabolism, lipid, iron

## Abstract

The p53 family of tumor suppressors, which includes p53, p63, and p73, has a critical role in many biological processes, such as cell cycle arrest, apoptosis, and differentiation. In addition to tumor suppression, the p53 family proteins also participate in development, multiciliogenesis, and fertility, indicating these proteins have diverse roles. In this review, we strive to cover the relevant studies that demonstrate the roles of p53, p63, and p73 in lipid and iron metabolism.

## Introduction

For over 40 years, p53 has been characterized as a master transcriptional regulator that mediates the expression of various genes to prevent aberrant cell growth (Ko and Prives, 1996). Just before the turn of the century, the *TP63* and *TP73* genes were discovered due to their significant homology to *TP53*, particularly in the DNA-binding domain (Kaghad et al., 1997; Schmale and Bamberger, 1997; Trink et al., 1998; Borremans et al., 2001). These three genes constitute the p53 family.

**FIGURE 1:**
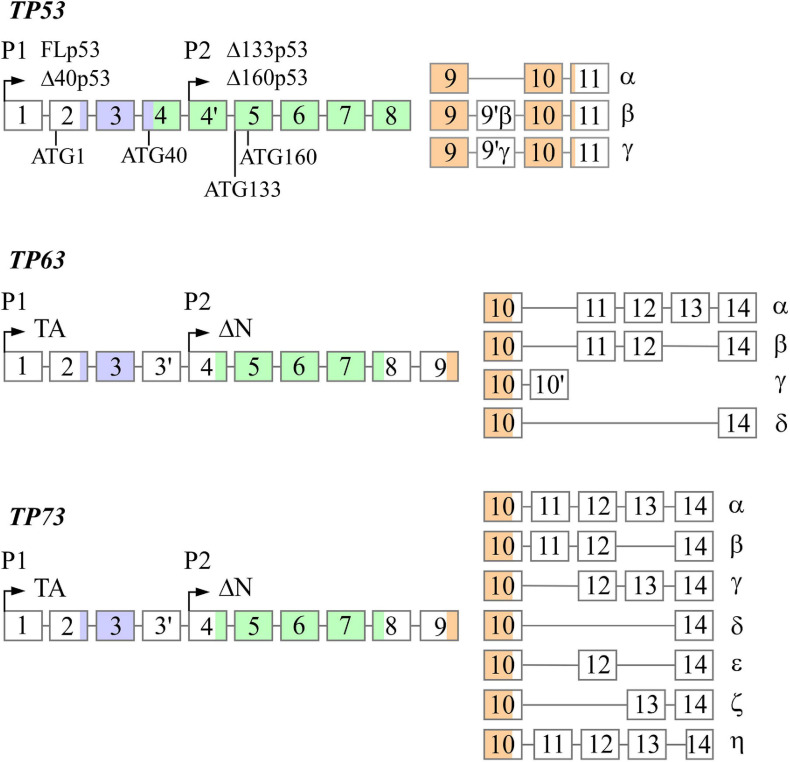
The genomic loci of *TP53*, *TP63*, and *TP73*. All three genes contain two promoters (P1 and P2) through which transcription initiation may occur, resulting in the formation of the N-terminal isoforms. *TP53* contains four alternative translation start sites termed ATG1, ATG40, AG133, and ATG160 that give rise to FLp53, Δ40p53, Δ133p53, and Δ160p53, respectively. Alternative splicing at the 3′ end produces the C-terminal isoforms in each gene with the exception of p63γ, which results from transcriptional termination in intron 10. There exists significantly homology between each gene in the transactivation domains (purple), DNA-binding domains (green), and oligomerization domains (orange).

The *TP53, TP63*, and *TP73* genes are expressed as multiple N- and C-terminal isoforms through two promoters and alternative splicing (Figure 1). In *TP53*, promoter 1 (P1) gives rise to two translation initiation start sites, termed ATG1 and ATG40, which produce full-length p53 (FLp53) and Δ40p53, respectively (Courtois et al., 2002; Yin et al., 2002). Both isoforms possess transactivation function even though Δ40p53 contains a truncated form of the conventional transactivation domain (Zhu et al., 1998; 2000; Harms and Chen, 2005). Similarly driven by P1, *TP63/73* express TAp63/73 isoforms, which have a transactivation domain that is comparable to the one found in FLp53 (Arrowsmith, 1999). By using promoter 2 (P2), all family members produce the N-terminally truncated isoforms, termed Δ133p53 and Δ160p53 in *TP53*, which arise from translation initiation start sites ATG133 and ATG160 (Bourdon et al., 2005), and ΔNp63/73 in *TP63/73* (Yang et al., 1998; 2000). Interestingly, despite lacking the conventional transactivation domain, ΔNp63 and ΔNp73 are transcriptionally active and can induce some p53 targets (Liu et al., 2004; Helton et al., 2006). Alternative splicing at the C-terminus of each gene generates additional isoforms. *TP53* produces three (α, β, γ) C-terminal isoforms (Bourdon et al., 2005), *TP63* produces four (α, β, γ, δ) C-terminal isoforms (Yang et al., 1998; Mangiulli et al., 2009), and *TP73* produces at least seven C-terminal isoforms (α, β, γ, δ, ε, ζ, η) (De Laurenzi et al., 1998; 1999). While the N-terminal isoforms of p53, p63, and p73 are well studied, the C-terminal isoforms remain largely uncharacterized.

The biological function of the p53 family proteins has been demonstrated through various mouse models. The very first p53-knockout model showed that mice deficient in p53 were prone to spontaneous tumors, but otherwise developed normally (Donehower et al., 1991). Later, it was discovered that p53 dysregulation, predominantly overexpression, can lead to impaired embryogenesis and other developmental defects (Luna et al., 1995; Sah et al., 1995; Parant et al., 2001; Zhang et al., 2012; Nostrand et al., 2014). Unlike p53, total p63-knockout mice exhibit severe epidermal and craniofacial abnormalities and die shortly after birth (Celli et al., 1999; Mills et al., 1999; Yang et al., 1999). Further studies revealed that ΔNp63 is responsible for the observed phenotype (Candi et al., 2006; Koster et al., 2007). In contrast, TAp63-knockout mice did not exhibit birth defects, but were prone to spontaneous tumors, indicating that TAp63 functions as a tumor suppressor to maintain genome stability (Suh et al., 2006; Guo et al., 2009; Su et al., 2009). Similarly, total p73-knockout mice were runty and had severe neurological defects, chronic inflammation, fertility issues (Yang et al., 2000), and impaired multiciliogenesis (Marshall et al., 2016; Nemajerova et al., 2016). It was later found that ΔNp73-knockout mice exhibited neurodegeneration (Wilhelm et al., 2010), whereas TAp73-knockout mice were prone to spontaneous tumors (Tomasini et al., 2008).

Continued research efforts into the more nuanced cancer-associated roles of the p53 family proteins is undeniably valuable. However, emerging evidence suggests that these proteins possess additional important functions that can affect various human diseases, such as diabetes mellitus and liver steatosis. This review will focus specifically on the roles of the p53 family in lipid and iron metabolism.

## Lipid Metabolism

Lipids play an important role in various biological processes and serve as an essential building block for many cellular structures. Tight regulation of lipid metabolism is crucial for proper organismal function, and dysregulation has been implicated in numerous diseases, such as Alzheimer’s disease and fatty liver disease (Hooijmans and Kiliaan, 2008; Hasson et al., 2016). There are three main sources of lipids: dietary lipids, fatty acids produced by hepatocytes and adipocytes, and lipoproteins produced by hepatocytes (Giammanco et al., 2015). In the lumen of the gastrointestinal tract, dietary lipids become emulsified by combining with bile salts, which allows for lipid hydrolysis and subsequent import to enterocytes (Hussain, 2014). In enterocytes, lipids are processed by the endoplasmic reticulum and packaged into lipoprotein bundles, called chylomicrons (Hussain, 2014; Giammanco et al., 2015), to allow for transport through the circulation (Alekos et al., 2020). Once chylomicrons arrive at a target cell, lipases break them down to permit cellular import of lipids (Alekos et al., 2020). Hepatocytes are then responsible for recycling chylomicron components to allow for later use (Alekos et al., 2020).

**FIGURE 2:**
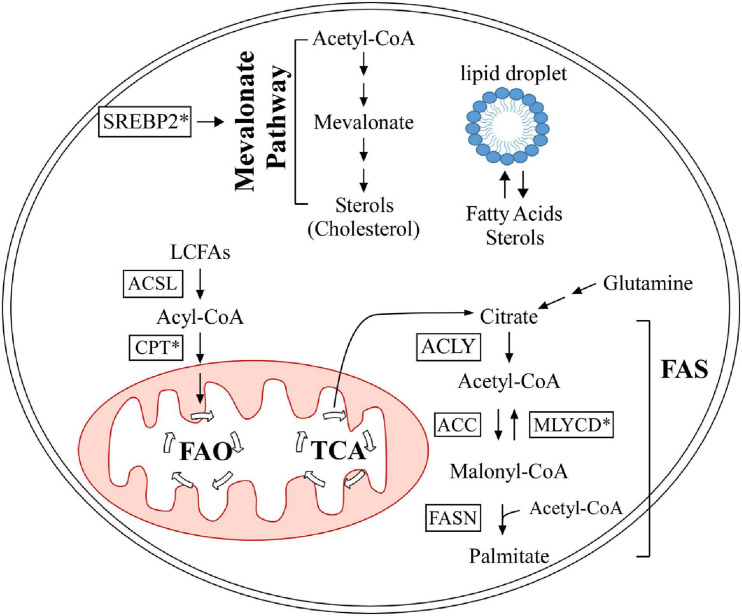
Summary of lipid metabolism, as it pertains to the p53 family. The mevalonate pathway is out lined in the upper half. Fatty acid oxidation and synthesis are portrayed in the lower half. Proteins marked with an asterisk are regulated by the p53 family. FAO, fatty acid oxidation; TCA, tricarboxylic acid cycle; FAS, fatty acid synthesis; ACSL, long-chain acyl-CoA synthetase; CPT, carnitine palmitoyltransferase; ACLY, ATP citrate lyase; MLYCD, malonyl-CoA decarboxylase; ACC, acetyl-CoA carboxylase; FASN, fatty acid synthase; SREBP2, sterol regulatory element-binding protein 2.

At the cellular level, lipids are categorized into three groups: structural lipids, lipid droplets, and bioactive lipids. Structural lipids are comprised of phospholipids and form cell and organelle membranes (Bohdanowicz and Grinstein, 2013), which are important for cellular compartmentalization. Lipids are also a main source of energy and are stored as modified sterols and fatty acids in specialized organelles called lipid droplets, which are predominantly found in adipocytes (Röhrig and Schulze, 2016; Olzmann and Carvalho, 2019). This modification gives sterols and fatty acids a neutral charge to form sterol esters (Korber et al., 2017) and triglycerides (Alves-Bezerra and Cohen, 2018), respectively. Bioactive lipids are unique in that they are involved in signal transduction and are categorized into multiple classes, including sphingolipids (Hannun and Obeid, 2008), diacylglycerols (Peter-Riesch et al., 1988), and eicosanoids (Levy et al., 2001). Sphingolipids are further categorized into several sub-classes, such as sphingomyelin, galactosylceramide, glucosylceramide, and sphingosine (Hannun and Obeid, 2018). Studies have shown that sphingolipids can modulate cell death and survival pathways, including apoptosis, cell growth/inhibition, and migration (Hannun and Obeid, 2018). Diacylglycerols serve as a secondary messenger in many critical cellular processes, such as neurotransmitter release (Ma et al., 2013) and insulin signaling in islet cells (Peter-Riesch et al., 1988). Eicosanoids have been implicated in mediating the inflammatory response (Levy et al., 2001). Lipids are exceedingly important for many cellular processes, from structure to signaling. In this review, we focus on the role of the p53 family proteins in cholesterol and fatty acid metabolism. Table 1 provides a summary of the p53 family targets that are involved in lipid metabolism, and Figure 2 briefly outlines cholesterol and fatty acid metabolism pathways.

**TABLE 1:** Targets of the p53 family that are associated with lipid metabolism.

Gene/protein target	Function	Regulation by p53 family member
SREBP-2	Upregulates mevalonate pathway genes	Down by p53 via ABCA1
*CAV1*	Promotes cellular cholesterol efflux	Up by p53
*DHRS3*	Promotes lipid droplet formation	Up by p53 and p63
*SOAT1*	Promotes cholesterol storage	Down by p53
*Cyp19*	Prevents cholesterol accumulation	Up by p53
*LIMA1*	Promotes cholesterol absorption in GI tract	Up by p53/p63/p73
*HMGCR, MVK, FDPS, FDFT1*	Promote mevalonate pathway	Up and Down by p53
*CrOT*	Transports byproducts of peroxisomal FAO to mitochondria	Up by p53
*Acad11*	Catalyzes first step of FAO	Up by p53
*MLYCD*	Converts malonyl-CoA to acetyl-CoA	Up by p53
*PANK1*	Catalyzes rate-limiting step of CoA production	Up by p53
*SIRT1*	Modulates histones and transcription factors to promote FAO	Up by p53 and p63
*LPIN1*	Upregulates FAO-associated genes	Up by p53
*CPT1C*	Transfers acyl group from long-chain fatty acyl to carnitine	Up by p53
*ADRB3*	Promotes lipolysis	Up by p53
*OPN*	Inhibits lipogenesis	Up by p53
*PGC1A/APLNR*	Inhibits FAO in cardiomyocytes	Up by p53
*SREBP-1c*	Promotes triglyceride synthesis and FAS	Down by p53
*ME1/ME2*	Converts malate to pyruvate, which produces NADPH	Down by p53
G6PD	Catalyzes first step of PPP, which produces NADPH	Down by p53 via protein-protein interaction
*TIGAR*	Promotes PPP activation	Up by p53
*PFKFB3*	Inhibits PPP activation	Down by p53
LKB1/AMPK	Pathway represses conversion of acetyl-CoA to malonyl-CoA	Pathway activated by p63
*CCDC3*	Inhibits FAS by binding hepatocyte receptors	Up by p63
*ATG5*	Promotes lipid droplet degradation	Up by p73

### p53

#### Cholesterol

Multiple studies have shown that p53 is implicated in regulating the levels of intracellular free cholesterol. Sterol Regulatory Element-Binding Protein 2 (SREBP-2) is a master transcriptional regulator of the mevalonate pathway and responds to sterol depletion by promoting cholesterol synthesis (Brown and Goldstein, 1997). It was found that p53 inhibits SREBP-2 maturation through the upregulation of *ABCA1* (Moon et al., 2019), an ATP-binding cassette transporter that inhibits cholesterol synthesis and drives cholesterol export when cholesterol stores are high (Yamauchi et al., 2015). Additionally, p53 can promote cholesterol export through the upregulation of *CAV1* (Bist et al., 2000), a scaffold protein that binds intracellular free cholesterol and facilitates its efflux (Fielding and Fielding, 1995). To enhance cellular cholesterol storage, p53 transactivates dehydrogenase/reductase member 3 (DHSR3) (Kirschner et al., 2010; Deisenroth et al., 2011), which decreases intracellular free cholesterol by increasing lipid droplet formation (Martin and Parton, 2006). Conversely, p53 has been shown to inhibit cholesterol storage by negatively regulating *SOAT1* (Oni et al., 2020). SOAT1 decreases intracellular free cholesterol by increasing cholesterol storage, thus disrupting the negative feedback loop that prevents cholesterol synthesis when free intracellular cholesterol levels are high (Oelkers et al., 1998). Furthermore, *Cyp19*, an aromatase essential for estrogen synthesis (Thompson and Siiteri, 1974), was found to be upregulated by p53, which prevents intracellular free cholesterol overload and adipocyte formation (Wang et al., 2013). One study revealed a potential link between p53 and *LIMA1*, also called *SREBP3*, in mediating cholesterol absorption in the gastrointestinal tract. p53 was shown to upregulate *LIMA1* through p53-response elements in its promoter (Ohashi et al., 2017), and LIMA1 promotes cholesterol absorption in the intestine (Zhang et al., 2018). Interestingly, there are some conflicting findings regarding p53 regulation of other mevalonate pathway genes. For example, one group showed that p53 inhibited expression of mevalonate pathway genes *HMGCR*, *MVK*, *FDPS*, and *FDFT1* (Moon et al., 2019), but another group showed that p53 enhanced expression of these genes (Laezza et al., 2015), suggesting that p53 may regulate some mevalonate pathway genes in a context-dependent manner. Collectively, these findings suggest that multiple targets are regulated by p53 to prevent intracellular free cholesterol accumulation and to maintain the integrity of the negative feedback loop that regulates cholesterol storage and synthesis.

#### Fatty Acids

Fatty acid oxidation, also known as β-oxidation and hereafter referred to as FAO, is the process of breaking down long-chain fatty acids (LCFAs), primarily in the mitochondria; FAO can be initiated in peroxisomes, but the byproducts undergo complete oxidation in the mitochondria (Qu et al., 2016). LCFAs are metabolized by long-chain acyl-CoA synthetase to form acyl-CoA (Mashek et al., 2007), which is then transported into the mitochondrial matrix by a series of reactions catalyzed by the carnitine palmitoyltransferase system (Rufer et al., 2009). Acyl-CoA is then used as a substrate to initiate FAO (Qu et al., 2016). Each cycle of FAO in the matrix removes two carbons from the fatty acid, until four carbons remain; these are then used to synthesize acetyl-CoA (Qu et al., 2016).

*De novo* fatty acid synthesis (FAS) is the process by which cells generate fatty acids that are used in various cellular processes (Röhrig and Schulze, 2016). FAS starts with citrate produced by the tricarboxylic acid (TCA) cycle or glutamine metabolism (Akram, 2014; Röhrig and Schulze, 2016). Citrate is then cleaved by ATP-citrate lyase to form acetyl-CoA, which is the starting substrate for FAS (Zaidi et al., 2012). Acetyl-CoA carboxylases convert acetyl-CoA to malonyl-CoA (Abu-Elheiga et al., 2000), at which point fatty acid synthase (encoded by *FASN*) catalyzes the reaction between seven malonyl-CoA molecules and one acetyl-CoA molecule to form palmitate, a long-chain fatty acid (Smith et al., 2003). Palmitate is then modified in length (Jakobsson et al., 2006) and degree of saturation (Igal, 2010) to form additional fatty acids.

p53 has been shown to predominantly promote FAO (Parrales and Iwakuma, 2016). RNA-seq analysis revealed that p53 upregulates *CrOT* (peroxisomal carnitine O-octanoyltransferase) (Goldstein et al., 2012), which is responsible for transporting byproducts of peroxisomal FAO to mitochondria to allow for complete oxidation (Longo et al., 2016). Similarly, another group showed that p53 pathway activation following γ-irradiation led to increased *CrOT* expression (Hage-Sleiman et al., 2017). In regards to mitochondrial FAO, p53 was found to upregulate *Acad11* (Jiang et al., 2015), which encodes acyl-CoA dehydrogenase and catalyzes the first step of FAO in the mitochondrial matrix (He et al., 2011). p53 can additionally promote FAO through upregulation of *MLYCD* (Liu et al., 2014), which encodes malonyl-CoA decarboxylase and converts the FAO inhibitor malonyl-CoA to acetyl-CoA (Foster, 2004). As evidenced by the name of many FAO intermediates, CoA is a critical molecule in many FAO reactions (Leonardi et al., 2005), and p53 was found to upregulate *PANK1*, which promotes CoA production (Wang et al., 2013). By complexing with FOXO3a, p53 transactivates *SIRT1* (Nemoto et al., 2004), a deacetylase that acts on histones and transcription factors to promote FAO (Rahman and Islam, 2011; Derdak et al., 2013). Activation of p53 in response to DNA damage and glucose starvation results in increased expression of *LPIN1*, a transcriptional co-activator, to promote FAO (Assaily et al., 2011). Lipin-1 also aids in diacylglycerol formation (Donkor et al., 2007), suggesting a role for p53 in diacylglycerol metabolism. There is evidence that p53 directly upregulates *CPT1C*, a neuron-specific carnitine palmitoyltransferase that transfers the acyl group from long chain fatty acyl to carnitine to initiate FAO (Lee and Wolfgang, 2012; Sanchez-Macedo et al., 2013). In addition to CPT1C, there are other tissue-specific carnitine palmitoyltransferase family members, such as CPT1a in the liver and CPT1b in muscle (Greenberg et al., 2009). Thus, it is possible that p53 might regulate FAO through CPT1a and CPT1b. p53 was shown to transactivate *ADRB3* (Kang et al., 2020), which promotes lipolysis, or the breakdown of triglycerides into fatty acids to allow for FAO (Arner and Langin, 2014). Interestingly, a p53 mutant could induce *ADRB3* to a higher degree (Kang et al., 2020). Likewise, studies showed that p53 can prevent lipogenesis through upregulation of *OPN*, which encodes osteopontin (Gómez-Santos et al., 2020). *In vivo* analyses in mouse livers showed that *OPN* levels were increased in response to an increase in p53 (Gómez-Santos et al., 2020). Conversely, a recent report showed that p53 inhibits FAO through *PGC1A* and *APLNR* in response to doxorubicin treatment in cardiomyocytes (Saleme et al., 2020). These data lead us to hypothesize that p53 could have tissue/cell-specific effects on FAO.

p53 has been shown to inhibit FAS (Parrales and Iwakuma, 2016). For example, p53 can negatively regulate transcription of SREBP-1c to inhibit FAS (Yahagi et al., 2003). SREBP-1c, a SREBP family member, is involved in triglyceride and fatty acid synthesis predominantly in the liver, which leads to fat accumulation (Shimano et al., 1997; Shimomura et al., 1998). Additionally, p53 has been implicated in inhibiting FAS through repression of NADPH production, a critical energy source utilized during FAS (Brose et al., 2016). p53 can inhibit NADPH production through negative transcriptional regulation of malic enzyme 1 and 2 (ME1 and 2) (Jiang et al., 2013). ME1/2 catalyze the formation of pyruvate from malate, which produces NADPH (Wise and Ball, 1964). Additionally, p53 prevents NADPH production through inhibition of glucose-6-phosphate dehydrogenase (G6PD) via protein-protein interaction, which requires p53’s C-terminus, DNA-binding domain, and tetramerization domain (Jiang et al., 2011). G6PD is an enzyme that catalyzes the first step of the pentose phosphate pathway (PPP), a major source of NADPH production (Ge et al., 2020). While reports show that p53 predominately inhibits NADPH production, some p53 targets have been identified as promoters of NADPH production. For example, TIGAR, a well-defined p53 target, activates PPP to drive NADPH production, which has been shown to prevent ROS formation (Bensaad et al., 2006). Additionally, it was found that p53 promotes NADPH production through suppression of PFKFB3 expression, which favors glycolysis over PPP (Franklin et al., 2016).

### p63 and p73

Several studies have unveiled an important role for p63 in lipid metabolism, although the mechanisms are not fully understood. It was shown that TAp63 deficiency in mice increases the incidence of obesity and liver steatosis and impairs FAO function (Su et al., 2012; Liao et al., 2017). It was found that TAp63 promotes FAO through upregulation of *SIRT1* (a previously described p53 target) and the LKBI/AMPK pathway, the latter of which prevents the conversion of acetyl-CoA to the FAO inhibitor malonyl-CoA (Li et al., 2020). As previously mentioned, p53 promotes the production of acetyl-CoA from malonyl-CoA (Liu et al., 2014), suggesting that the p53 family can transactivate multiple targets to prevent malonyl-CoA formation. Additionally, TAp63 was shown to inhibit FAS by upregulating *CCDC3* (Liao et al., 2017), which encodes a soluble protein that binds to hepatocyte receptors (Kobayashi et al., 2010). While there is limited research on p63 and cholesterol regulation, it has been shown that p63, like p53, inhibits cellular cholesterol accumulation through *DHSR3* (Kirschner et al., 2010) and promotes intestinal cholesterol absorption through *LIM1A* (Zhang et al., 2018).

Phenotypic similarities between p63- and p73-deficient mice suggest that p73 has an analogous role in regulating lipid metabolism. In response to nutrient deprivation, loss of p73 leads to lipid accumulation in mouse livers (He et al., 2013). Mechanistically, TAp73α/β were shown to modulate lipid metabolism through *ATG5*, a gene that is necessary for autophagy (He et al., 2013). Autophagy is an intracellular process that, among other things, can break down lipid droplets to allow for FAO (Ye et al., 2018; Saito et al., 2019). As such, gene transfer of *ATG5* to p73-knockout mice mitigated the accumulation of lipid droplets in the mouse livers (He et al., 2013). As with p53 and p63, p73β can upregulate *LIM1A* to increase cholesterol absorption (Y. Y. Zhang et al., 2018). These data suggest that p73 prevents lipid accumulation through a mechanism that is quite different from how p53 and p63 regulate this process.

## Iron Metabolism

**FIGURE 3:**
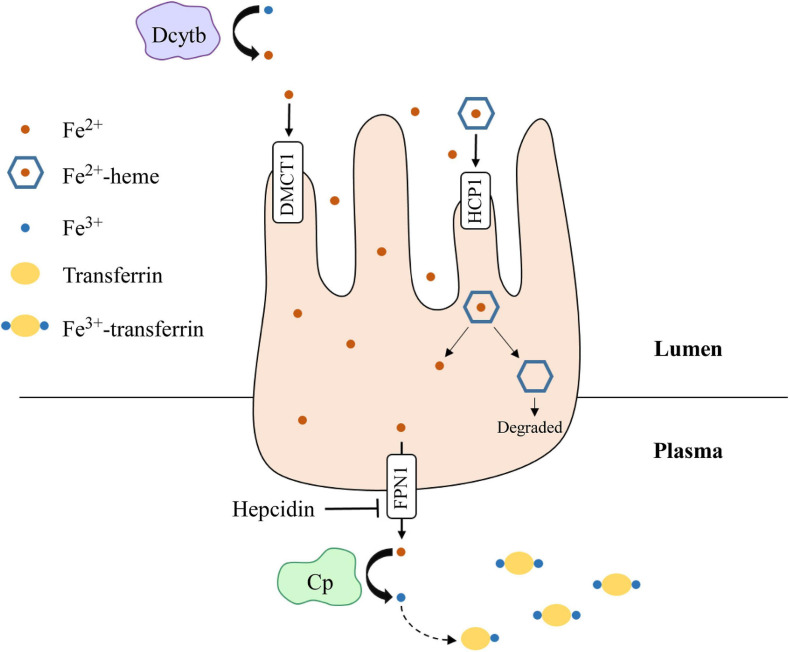
Dietary iron absorption and transport from enterocytes to the plasma. Fe^3+^ is reduced to Fe^2+^ via duodenal cytochrome b (Dcytb) in the intestinal lumen. Non-heme-iron is transported into enterocytes via divalent metal cation transporter 1 (DMCT1), while heme-iron is transported through haem carrier protein 1 (HCP1). Heme dissociates from iron and is degraded. Fe^2+^ is then exported out of enterocytes by ferroportin 1 (FPN1), whose function is inhibited by hepcidin. Once in the plasma, Fe^2+^ is oxidized to Fe^3+^ by ceruloplasmin (Cp), which then binds transferrin for transport through the plasma. Iron is transported into target cells for storage or use.

Iron is an essential element for all living entities and plays an important role in many cellular processes, such as oxygen transport and cell proliferation (Kim and Nemeth, 2015; Wallace, 2019). Additionally, iron is a critical cofactor that is required for various metabolic activities, such as DNA synthesis (Puig et al., 2017). An organism’s main source of iron is through dietary intake (Waldvogel-Abramowski et al., 2014). In the gastrointestinal tract, iron exists as non-heme- or heme-iron (Waldvogel-Abramowski et al., 2014); heme is a porphyrin that contains iron (Fiorito et al., 2020). At physiological pH, non-heme iron is present in the ferric (Fe^3+^) state, but cells can only absorb it in the ferrous (Fe^2+^) state (Wallace, 2019). Duodenal cytochrome B (Dcytb) reduces Fe^3+^ to Fe^2+^ in the lumen and ferrous iron is absorbed by enterocytes through divalent metal cation transporter 1 (DMCT1) (Wallace, 2019). On the other hand, heme-iron is directly imported by haem carrier protein 1 (HCP1). Once in enterocytes, iron is exported by ferroportin 1 (FPN1), whose function is inhibited by hepcidin (Waldvogel-Abramowski et al., 2014). In the plasma, Fe^2+^ is converted back to Fe^3+^ by ceruloplasmin and binds to transferrin for transport through the circulation (Attieh et al., 1999). A summary schematic of this process is shown in Figure 3. Once iron has entered the target cell, it binds ferritin until it is needed (Waldvogel-Abramowski et al., 2014). Regulation of iron metabolism is exceedingly important because iron overload, like in hemochromatosis (Bacon et al., 2011), can lead to heart disease and liver cirrhosis, while deficiency can result in anemia and developmental impairments (Abbaspour et al., 2014). The p53 family has been implicated in mediating iron metabolism to prevent iron dysregulation.

### p53

Iron metabolism exerts regulatory functions over p53 and in turn, p53 can regulate iron metabolism. Excess iron leads to decreased p53 expression (Shen et al., 2014), whereas iron depletion leads to p53 accumulation (Liang and Richardson, 2003; Kim et al., 2007). Additionally, direct binding of heme to p53 protein inhibits p53 transcriptional activity and possibly promotes p53 degradation (Shen et al., 2014). As such, a feedback loop between iron and p53 exists wherein iron overload inhibits p53 activity and p53 inhibits iron accumulation. At the systemic level, p53 upregulates *HAMP* (encoding hepcidin) to inhibit iron efflux from enterocytes (Weizer-Stern et al., 2007) and thus, prevents iron from entering the circulation when it is not needed. To prevent iron overload at the cellular level, p53 directly transactivates several targets, including *FXN* (frataxin) (Shimizu et al., 2014), *FDXR* (ferredoxin reductase) (Hwang et al., 2001; Liu and Chen, 2002), and *ISCU* (iron-sulfur cluster assembly enzyme) (Funauchi et al., 2015). Frataxin is an iron binding protein that regulates mitochondrial iron homeostasis to prevent iron overload (Cavadini et al., 2000) and thus, p53 upregulates frataxin to inhibit mitochondrial iron overload. Additionally, frataxin is necessary for iron-sulfur cluster (ISC) biogenesis (Shimizu et al., 2014) and ISCs are critical for mitochondrial function (Shimizu et al., 2014). In addition to aiding in electron transport during redox reactions (Johnson et al., 2005), ISCs serve as a co-factor for many essential enzymes (Baranovskiy et al., 2012). We also showed that p53 regulates mitochondrial iron metabolism through a FDXR-p53 loop (Liu and Chen, 2002; Zhang et al., 2017). FDXR plays a critical role in ISC biogenesis and steroid hormone synthesis by transferring electrons from NADPH to ferredoxin 1 and 2 (FDX1 and 2) (Brandt and Vickery, 1992; Sheftel et al., 2010). p53 drives the FDXR-p53 loop to upregulate FDXR, which then transfers electrons to FDX2, ultimately preventing iron overload at the cellular level (Zhang et al., 2017). Furthermore, p53 upregulates ISCU, which increases translation of ferritin heavy chain mRNA (*FTH1*) and destabilizes transferrin receptor mRNA (*TFRC*) (Funauchi et al., 2015), therefore increasing cellular iron storage and decreasing cellular iron import. p53 can also regulate iron metabolism through post-transcriptional modifications of Iron Regulatory Protein 1 and 2 (IRP1 and IRP2) (Zhang et al., 2008). IRP1/2 alter the expression of proteins associated with iron transport and storage by binding to conserved iron-regulatory elements (IRE) in target mRNAs (Volz, 2008). Interestingly, the binding of IRP1/2 to a target mRNA has context-dependent outcomes, wherein binding can promote both mRNA degradation and mRNA translation (Volz, 2008). Studies showed that overexpression of p53 led to reduced IRP1 and 2 activity, resulting in increased translation of ferritin mRNA and decreased stability of transferrin receptor mRNA (Zhang et al., 2008). This regulation ultimately leads to an increase in cellular iron stores and a decrease in cellular iron import.

Ferroptosis is a specific form of iron-mediated cell death in which oxidative stress from reactive oxygen species (ROS) leads to the formation of lipid peroxides and accumulation of lipid peroxides triggers the ferroptotic response (Dixon et al., 2012; Lu et al., 2018). Iron has a critical role in promoting ROS formation through several mechanisms. First, iron functions as a co-factor for enzymes that catalyze the formation of ROS (Dixon and Stockwell, 2014). In addition, Fe^2+^ reacts with hydrogen peroxide through the Fenton reaction, resulting in the production of free radicals, a potent form of ROS (Wardman and Candeias, 1996). ROS can then promote lipid peroxidation of cellular membranes, which leads to compromised membrane integrity and cellular damage (Yin et al., 2011). However, several intracellular reducing pathways have been found to block ROS and subsequent accumulation of lipid peroxides (Lu et al., 2018). Import of cystine into the cell via system x_*c*_^–^ (encoded by *SCL7A11*) ultimately results in the synthesis of glutathione, a strong antioxidant (Lu et al., 2018; Sato et al., 2018). GPX4, a member of the glutathione peroxidase family, uses glutathione as a co-activator to reduce lipid peroxides, thus preventing ferroptosis (Lu et al., 2018). Ferroptosis has been implicated in a variety of diseases, such as cell death during ischemia (Gao et al., 2015) and neurodegeneration in Alzheimer’s disease (Masaldan et al., 2019). Interestingly, p53 can promote and inhibit ferroptosis in a context-dependent manner (Liu et al., 2020). For example, p53 is able to inhibit ferroptosis through p21, a primary p53 target that inhibits glutathione degradation (Tarangelo and Dixon, 2018). As such, upregulation of p21 by p53 inhibits glutathione degradation and promotes GPX4 activity (Tarangelo and Dixon, 2018). p53 was also shown to prevent ferroptosis by promoting the nuclear, but inhibiting the plasma membrane, accumulation of dipeptidyl-peptidase 4 (DPP4) (Xie et al., 2017). DPP4 in the nucleus upregulates *SLC7A11*, leading to increased GPX4 function and subsequent inhibition of ferroptosis (Xie et al., 2017). Interestingly, p53 can promote ferroptosis by directly inhibiting *SLC7A11* expression (Jiang et al., 2015). Additionally, p53 promotes ferroptosis through upregulation of *SAT1*, which facilitates the production of lipid peroxides (Ou et al., 2016). A recent study showed that Mdm2 and Mdm4 can induce ferroptosis (Venkatesh et al., 2020). Since Mdm2 is a target of p53, it is possible that p53 can act through Mdm2/4 to modulate the induction of ferroptosis. The role of both wild-type and mutant p53 in ferroptosis was discussed comprehensively in a recent review (Liu et al., 2020).

### p63 and p73

Recent evidence suggests an important role for p63 and p73 in iron metabolism. Like p53, p63, and p73 can be destabilized by an excess of heme (Shen et al., 2014). Conversely, iron depletion was found to stabilize p73, and possibly p63, to promote apoptosis and cell cycle arrest in a p53-independent manner (Calabrese et al., 2020). These data suggest that iron overload inhibits, whereas iron depletion promotes, p63 and p73 activity, which is similar to the effect of iron overload and depletion on p53. Recent studies in our lab revealed a potential mechanism through which iron overload can influence p63/p73 mRNA stability and protein expression. We showed that TAp63 expression can be repressed by IRP2 and likewise, IRP2 deficiency lead to increased expression of TAp63 (Zhang et al., 2020). Additionally, we showed that IRP2 binds to the IRE in p63 mRNA to regulates its stability (Zhang et al., 2020). Similarly, we found that FDXR regulates p73 mRNA stability through IRP2 (Zhang et al., 2020). These observations represent an important step in understanding how iron metabolism regulates p63 and p73.

Several lines of evidence suggest a role for p63 and p73 in mediating ferroptosis. For example, ferroptosis has been shown to promote liver steatosis and inflammation (Tsurusaki et al., 2019). We and others found that p63-deficient mice were prone to liver steatosis (Jiang et al., 2018). Additionally, both p63- and p73-deficient mice exhibited a high degree of liver inflammation (Jiang et al., 2018; Zhang et al., 2020). Moreover, before the term ferroptosis was coined, we found that p63 inhibited cell death caused by oxidative stress through GPX2 (Yan and Chen, 2006), a member of the same phospholipid peroxidase family as GPX4 (Chu, 1994). Aforementioned, ferroptosis ensues when the cell is unable to overcome oxidative stress. Another study revealed that ΔNp63 promoted glutathione metabolism, thus permitting GPX4 function and inhibiting the ferroptotic pathway (G. X. Wang et al., 2017). These findings suggest that p63 regulates ferroptosis through multiple glutathione peroxidase family members. As previously mentioned, p63 activates the LKB1/AMPK pathway and a group recently showed that this pathway inhibits ferroptosis (Li et al., 2020). While the role of p73 in ferroptosis is less studied, one report showed that TAp73-knockout mouse embryonic fibroblasts were particularly prone to oxidative stress (Agostini et al., 2016). Another study showed that TAp73 is able to mitigate the effect of oxidative stress on mitochondrial integrity (Marini et al., 2018). These data suggest a role for TAp73 in suppressing ferroptosis.

## Future Directions

There is growing evidence that, in addition to mediating tumor suppression, the p53 family plays an important role in lipid and iron metabolism. However, there is a need for more research on these critical topics. It would be of interest to further explore how p53 is involved in regulating bioactive lipids. Additionally, it would be worthwhile to delve into the mechanisms by which p63/p73 regulate lipid and iron metabolism. While there is evidence that aberrant iron metabolism affects lipid metabolism and ferroptosis, how p53 engages lipid and iron metabolism in ferroptosis needs to be further explored. Moreover, several fundamental questions remain unanswered: Can p63 and p73 function independently of p53 in both lipid and iron metabolism? How does regulation of lipid and iron metabolism differ between the N- and C-terminal isoforms of each protein? Does regulation of lipid and iron metabolism by the p53 family contribute to common diseases associated with these processes, such as diabetes or anemia? Finally, can the p53 family proteins themselves, or the pathways regulated by the p53 family, be manipulated to ameliorate the effect of lipid or iron dysregulation on pathogenesis of diabetes and other diseases? A comprehensive understanding of how the p53 family mediates lipid and iron metabolism will likely provide an insight into the pathways that drive various human diseases.

## Author Contributions

KL, JZ, and XC wrote the article. All authors contributed to the article and approved the submitted version.

## Conflict of Interest

The authors declare that the research was conducted in the absence of any commercial or financial relationships that could be construed as a potential conflict of interest.

## Publisher’s Note

All claims expressed in this article are solely those of the authors and do not necessarily represent those of their affiliated organizations, or those of the publisher, the editors and the reviewers. Any product that may be evaluated in this article, or claim that may be made by its manufacturer, is not guaranteed or endorsed by the publisher.
